# Novel Method for Determining Standard Enthalpy and Entropy of Volatilisation of Chromia Exposed to Humidified Oxygen at 298 K Based on Transport Theory of Multicomponent Gas Mixtures

**DOI:** 10.3390/e27020101

**Published:** 2025-01-21

**Authors:** Thammaporn Thublaor, Watcharapon Tengprasert, Grid Suparapinyopapkul, Thanasak Nilsonthi, Walairat Chandra-ambhorn, Somrerk Chandra-ambhorn

**Affiliations:** 1 High Temperature Corrosion Research Centre, Department of Materials and Production Technology Engineering, Faculty of Engineering, King Mongkut’s University of Technology North Bangkok, 1518, Pracharat 1 Road, Bangsue, Bangkok 10800, Thailand; thammaporn.t@eng.kmutnb.ac.th (T.T.); watcharapon.t@email.kmutnb.ac.th (W.T.); grid.s@email.kmutnb.ac.th (G.S.); thanasak.n@eng.kmutnb.ac.th (T.N.); 2 Department of Chemical Engineering, School of Engineering, King Mongkut’s Institute of Technology Ladkrabang, Chalongkrung 1 Road, Lat Krabang, Bangkok 10520, Thailand; walairat.ch@kmitl.ac.th

**Keywords:** chromium poisoning, volatilisation, chromia, interconnect, solid oxide fuel cells

## Abstract

Based on the transport theory of multicomponent gas mixtures, we proposed an expression for determining the standard volatilisation enthalpy and entropy of chromia exposed to humidified oxygen at 298 K. The use of this expression could lead to the quantification of those thermodynamic quantities when the mass flux of Cr loss due to the volatilisation of chromia is determined. We thus experimentally measured the mass fluxes of Cr loss due to volatilisation in humidified atmospheres at 873–1073 K. As a result, the standard enthalpy and entropy of the volatilisation of chromia subjected to humidified oxygen at 298 K were quantified, giving the values of 61.1 ± 1.0 kJ mol^−1^ and −43.0 ± 1.9 J K^−1^ mol^−1^, respectively. The measured and calculated mass fluxes of Cr loss due to volatilisation tended to be related to absolute temperature in the Arrhenius-like form. We proposed the use of the line of the mass flux of Cr loss due to volatilisation in an Arrhenius-like diagram, which was shifted by the different thermodynamic data used to graphically assess the reliability of the thermodynamic data obtained from this work and the literature.

## 1. Introduction

To contribute to the achievement of the sustainable development goal on the use of clean energy, high-temperature solid oxide fuel cells (SOFCs) have been developed to convert chemical energy to electrical energy by the use of hydrogen as a fuel with water vapour as a product [1,2,3,4]. These cells principally consist of an anode, electrolyte and cathode and a part called an interconnect to separate the cathode from the anode sides. Current SOFC development aims at enhancing cell performance by lowering the operating temperature to 1073 K or lower [5,6,7,8,9,10]. At these temperatures, ferritic stainless steels have been widely investigated as interconnects instead of ceramic interconnects primarily because of their relatively cheap cost and thermal expansion coefficient that is compatible with an SOFC single cell [10,11,12,13,14,15,16,17,18]. However, constituent elements in stainless steels, particularly Cr and Mn, can be oxidised under SOFC environments to form thermally grown oxides consisting of Cr_2_O_3_ with MnCr_2_O_4_ on the top [10,18,19]. These oxides are reactants for producing Cr-containing volatile species [20,21,22,23,24], which can further diffuse to the cathode and present there as contaminates [11,19,25,26,27]. This poisoning effect was reported to be a major barrier to the mitigation of SOFC stack degradation [28,29].

To predict the chromium poisoning effect correctly, reliable thermodynamic data on the species involved in volatilisation reactions are needed [24]. Opila et al. investigated Cr species volatilisation [22,24]. They applied a transpiration method to measure the mass flux of Cr loss due to the volatilisation of Cr_2_O_3_ at different temperatures and water vapour contents [22]. Thermodynamic analysis, particularly the Van’t Hoff equation, was used to help determine the enthalpy and entropy of volatilisation at high temperatures [22]. Recently, quantum calculations were applied by Bauschlicher et al. to determine thermodynamic data concerning volatilisation [24]. Apart from thermodynamic and quantum theory aspects, Young and Pint [30] introduced the concepts of transport theory and gas kinetics, particularly the Chapman–Enskog model, to be applied in Cr volatilisation estimation. They calculated the mass flux of Cr loss due to the volatilisation of Cr_2_O_3_ exposed to humidified air by treating the bulk gas as a binary gas mixture [30]. Inspired by the work of Young and Pint [30], we extended the concept of gas transport to a multicomponent gas mixture and proposed an expression which shows the mass flux of Cr loss due to volatilisation as a function of standard volatilisation enthalpy and entropy at high temperature [31]. However, it was derived under the assumption that the enthalpy and entropy of the reaction were unchanged with temperature [31], analogous to the Ellingham approximation for oxidation reaction [32]. In addition, the thermal oxide scale studied in that work [31] contained both chromia and Mn-Cr spinel thermally grown on stainless steel. Based on that work, we proposed here a method for determining the standard volatilisation enthalpy and entropy only of chromia exposed to humidified oxygen at 298 K, which could lead to the determination of the standard enthalpy of formation of the volatile species, i.e., CrO_2_(OH)_2_, and the molar entropy of that species at 298 K. Such a determination also took into account the changes in the enthalpy and entropy of reaction with temperature. This highlights that while previous works applied thermodynamic concepts [22] or quantum calculations [24] to determine thermodynamic data concerning Cr species volatilisation, the present work took another approach—the transport theory—to determine the standard volatilisation enthalpy and entropy at 298 K. The reliability of thermodynamic data was assessed by using such data to calculate the mass flux of Cr loss due to volatilisation and compare it with the Cr loss flux measured from the experiment and with the mass flux of Cr loss due to volatilisation calculated using another data set [22,24,33].

In addition, in order to determine the enthalpy and entropy of the reaction, the measurement of chromium volatilisation at different temperatures was carried out. It was experimentally observed that the mass flux of Cr loss due to volatilisation from Fe-16Cr (in wt %) [34] and Fe-12Cr [31] exposed to humidified oxygen tends to be Arrhenius-like. The explanation of this behaviour has been discussed recently under the Ellingham approximation. In this paper, such an approximation was removed in order to give a more complete explanation for the observed Arrhenius-like relationship between the mass flux of Cr loss due to volatilisation and absolute temperature.

## 2. Theoretical Background

At high temperatures, chromium oxide can react with oxygen and water vapour, resulting in the formation of volatile chromium species. In humidified oxygen at 873–1073 K, the dominant volatile species in humidified oxygen was reported to be CrO_2_(OH)_2(g)_, which can be formed by Reaction (1) [22,30,31].

(1) $$\frac{1}{2}{\mathrm{Cr}}_{2}O_{3(s)}+\frac{3}{4}O_{2(g)}{+H}_{2}O_{\left(g\right)}{=\mathrm{CrO}}_{2}{\left(\mathrm{OH}\right)}_{2(g)}$$

**Figure 1:**
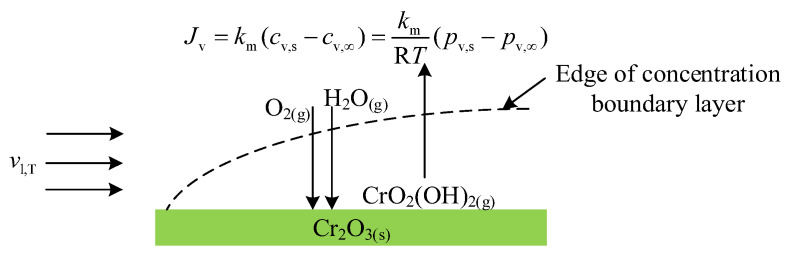
Transport phenomena of gases taking part in Reaction (1).

The thermodynamic data corresponding to Cr species volatilisation reactions were conventionally determined from either thermodynamic concepts [22] or quantum calculations [24], as previously mentioned. However, considering the transport phenomena of gases taking part in Reaction (1) expressed in Figure 1, thermodynamic data, i.e., $\Delta_{r}H_{(1),298K}^{\circ }$ and $\Delta_{r}S_{(1),298K}^{\circ }$, could also be determined using transport theory.

As shown in Figure 1, when the gas mixture containing water vapour and oxygen passes through the surface of the Cr_2_O_3_ pellet, a momentum boundary layer is built up, whereas the concentration boundary layer is built up due to Reaction (1). When H_2_O and O_2_ contact the surface of the Cr_2_O_3_ solid, Reaction (1) occurs, producing Cr volatile species, CrO_2_(OH)_2_. As H_2_O and O_2_ are consumed by the reaction at the Cr_2_O_3_ surface, concentrations of H_2_O and O_2_ are lower than those in the bulk stream, introducing a concentration gradient across the solid surface and the bulk gas. Meanwhile, CrO_2_(OH)_2_ is produced at the surface of Cr_2_O_3_, with the concentration higher than in the bulk gas, and transfers from the Cr_2_O_3_ surface to the bulk gas stream due to its concentration difference. Gas transport in this manner is called the convective mass transfer flux. The molar mass flux of CrO_2_(OH)_2_ transferring across the concentration boundary layer ($J_{v}$) (mol cm^−2^ s^−1^) can be determined by the equation analogous to Newton’s law of cooling [35], where the subscript v stands for CrO_2_(OH)_2_.

(2) $$J_{v}=k_{m}\left(c_{v,s}-c_{v,\infty }\right)$$

In this equation, $k_{m}$ is defined as the convective mass transfer coefficient (cm s^−1^). $c_{v,s}$ and $c_{v,\infty }$ are the molar concentrations of CrO_2_(OH)_2_ (mol cm^−3^) at the surface of Cr_2_O_3_ and in the bulk gas, respectively. For the ideal gas mixture, a molar concentration of each gaseous species (c) can be determined by using the ideal gas law, i.e., c=p/RT, where p is the partial pressure of that species (bar), R is the universal gas constant (83.1451 bar cm^3^ mol^−1^ K^−1^) and T is the absolute temperature (K). By turning the molar concentration into the partial pressure (p) using the ideal gas law, the CrO_2_(OH)_2_ flux can be expressed by Equation (3), where $p_{v,s}$ and $p_{v,\infty }$ are the partial pressures of the CrO_2_(OH)_2_ at the solid surface of Cr_2_O_3_ and in the bulk gas, respectively.

(3) $$J_{v}=\frac{k_{m}}{RT}\left(p_{v,s}-p_{v,\infty }\right)$$

According to Reaction (1), one mole of CrO_2_(OH)_2_ consumes one mole of Cr from Cr_2_O_3_. Thus, the molar mass flux of CrO_2_(OH)_2_ equals the molar mass flux of Cr loss due to volatilisation. In addition, the partial pressure of the volatile species in the bulk gas is considered negligible in comparison to that at the solid surface of Cr_2_O_3_ [30]. Thus, Equation (3) may be simplified and presented in the form of the mass flux of Cr loss due to volatilisation ($j_{\mathrm{Cr}}$) as follows.

(4) $$j_{\mathrm{Cr}}=\frac{M_{\mathrm{Cr}}}{RT}k_{m}p_{v,s}$$

The estimation of the mass transfer coefficient ($k_{m}$) of a gaseous species in the multicomponent gas mixture is given in standard references [36,37], including our recent work [31]. Such a method can be applied for the system comprising a dilute volatile species v (CrO_2_(OH)_2_) and carrier gases (Ar, O_2_ and H_2_O) as the $k_{m}$ value can be obtained by Equation (5), where $D_{v\text{-}\mathrm{mix}.}$ is the diffusivity of species v through the gas mixture, $v_{g}$ is the kinematic viscosity, $v_{l,T}$ is the linear gas velocity at the temperature T and L is the sample length [30,31].

(5) $$k_{m}=0.664\;\frac{D_{v-\mathrm{mix}.}^{2/3}}{v_{g}^{1/6}}{\left(\frac{v_{l,T}}{L}\right)}^{1/2}$$

$D_{v-\mathrm{mix}.}$ can be calculated from Equation (6) [37], where $y_{A}$ is the mole fraction of the species A, which can be Ar, O_2_ or H_2_O.

(6) $$\frac{1}{D_{v-\mathrm{mix}.}}=\frac{y_{\mathrm{Ar}}}{D_{v-\mathrm{Ar}}}+\frac{y_{O_{2}}}{D_{v-O_{2}}}+\frac{y_{H_{2}O}}{D_{v-H_{2}O}}$$

Hirschfelder et al. [35,38] proposed the binary gas diffusivity, as presented in Equation (7) for the diffusion coefficient of the binary gases comprising the volatile species v and the gas A ($D_{v-A}$). $D_{v-A}$ can be estimated using Equation (7) [30], where T is the absolute temperature (K); $P_{T}$ is the total pressure of the gas mixture, which is 1 atm for the present study; and M is the molar mass of the interested species (g mol^−1^). $\sigma_{v-A}$ is the collision diameter of the v–A binary pair (Å) [35] estimated as the average of the collision diameter of species v ($\sigma_{v}$) and that of species A ($\sigma_{A}$) [30]. $\Omega_{D,v-A}$ is the collision integral for diffusion [36], which is a function of $k_{B}T/\varepsilon_{v-A}$, where $k_{B}$ is the Boltzmann constant and $\varepsilon_{v-A}$ is the characteristic energy for molecular interaction for the v–A binary gas mixture [35]. $\varepsilon_{v-A}$ is estimated as the square root of the multiplication of the characteristic energy of species v ($\varepsilon_{v}$) and that of species A ($\varepsilon_{A}$) [30].

(7) $$D_{v-A}=\frac{1.858\times 10^{-3}\cdot T^{1.5}}{\sigma_{v-A}^{2}\Omega_{D,v-A}\cdot P_{T}}\sqrt{\frac{1}{M_{v}}+\frac{1}{M_{A}}}$$

Table 1 reports the molar masses (M), the collision diameters (σ) and the characteristic energies divided by the Boltzmann constant ($\varepsilon /k_{B}$) of CrO_2_(OH)_2_, Ar, O_2_ and H_2_O [30,37,39]. With the known $k_{B}T/\varepsilon_{v-A}$ value, $\Omega_{D,v-A}$ can be determined from an empirical formula given by Neufeld et al. [36,40].

The kinematic viscosity of the gas mixture ($v_{g}$) is defined as its viscosity ($\mu_{\mathrm{mix}}$) divided by its density ($\rho_{\mathrm{mix}}$) described by Equation (8) [30,31]. $\rho_{\mathrm{mix}}$ can be determined using Equation (9). $\mu_{\mathrm{mix}}$ can be obtained from Equation (10), where the right-hand side of this equation consists of viscosities of the carrier gases (Ar, O_2_ and H_2_O) with their coefficients (κ) [36]. The viscosity of gas A ($\mu_{A}$) can be obtained from Equation (11) [30,31], where $\Omega_{\mu ,A}$ is the collision integral for viscosity of gas A [31,36], which empirically relates to $k_{B}T/\varepsilon_{A}$ by the formula proposed Neufeld et al. [36,40]. $\kappa_{A\text{-}\mathrm{mix}}$ is the coefficient for gas A in the gas mixture. Its value can be determined by Equation (12), where the right-hand side of this equation contains mole fractions of the carrier gases and parameters ϕ. The latter parameter can be obtained for the pair of gases λ and ω ($\phi_{\lambda \text{-}\omega }$) by Equation (13) [31,36].

(8) $$v_{g}=\frac{\mu_{\mathrm{mix}}}{\rho_{\mathrm{mix}}}$$

(9) $$\rho_{\mathrm{mix}}={(M}_{\mathrm{Ar}}y_{\mathrm{Ar}}{+M}_{O_{2}}y_{O_{2}}+M_{H_{2}O}y_{H_{2}O})\frac{P_{T}}{RT}$$

(10) $$\mu_{\mathrm{mix}}=\kappa_{\mathrm{Ar}\text{-}\mathrm{mix}}\mu_{\mathrm{Ar}}+\kappa_{O_{2}\text{-}\mathrm{mix}}\mu_{O_{2}}+\kappa_{H_{2}O\text{-}\mathrm{mix}}\mu_{H_{2}O}$$

(11) $$\mu_{A}=\frac{2.6693\times 10^{-5}\sqrt{M_{A}T}}{\sigma_{A}^{2}\Omega_{\mu ,A}}$$

(12) $$\kappa_{A\text{-}\mathrm{mix}}=\frac{y_{A}}{y_{\mathrm{Ar}}\phi_{A-\mathrm{Ar}}+y_{O_{2}}\phi_{{A\text{-}O}_{2}}+y_{H_{2}O}\phi_{{A\text{-}H}_{2}O}}$$

(13) $$\phi_{\lambda -\omega }=\frac{1}{\sqrt{8}}\cdot \frac{{\left(1+\frac{{\left(\mu_{\lambda }/\mu_{\omega }\right)}^{1/2}}{{\left(M_{\lambda }/M_{\omega }\right)}^{1/4}}\right)}^{2}}{{\left(1+\left(M_{\lambda }/M_{\omega }\right)\right)}^{1/2}}$$

The $v_{l,T}$ value can be obtained by Equation (14) [31]. With the known $D_{v-\mathrm{mix}.}$, $v_{g}$ and $v_{l,T}$, the $k_{m}$ according to Equation (5) can be obtained.

(14) $$v_{l,T}=\frac{v_{l,298\;K}}{298}\cdot T$$

## 3. Experimental Section

The samples used in this work were chromia pellets made from chromium oxide (Cr_2_O_3_) powder with a purity of about 99% provided by CARLO ERBA (Emmendingen, Germany) reagents. The powder was mixed with deionised water, which served as binder. The powder was further compressed in a 2 cm diameter cylindrical mould using a hydraulic machine with a compression of 80 kg cm^−2^ for 4 min. The chromia pellets were sintered at 1573 K in air for 4 h, counting when the set-up temperature was reached using the average heating rate of 287.17 K min^−1^. The average cooling rate was 277.16 K min^−1^ from 1573 K to 1073 K, and then the pellet was further slowly cooled down to room temperature. The pore size distribution analyser (BELSORP-mini) (Microtrac, Osaka, Japan) was performed giving the result that the pellet had a density of about 97% of the theoretical value.

**Figure 2:**
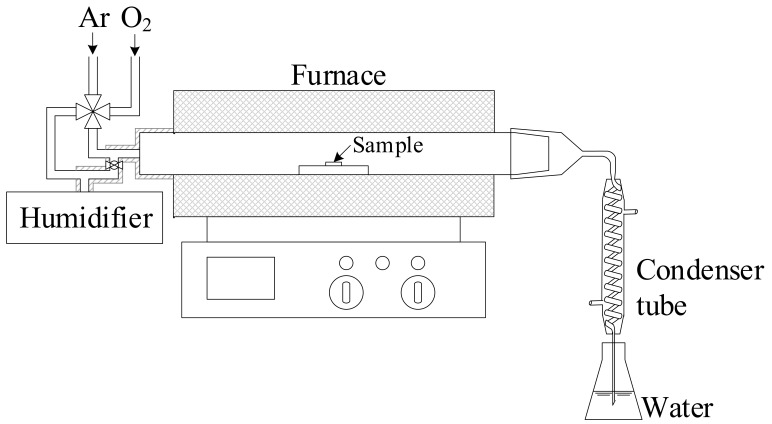
Schematic sketch of the Cr volatilisation measurement set-up.

Figure 2 shows a schematic sketch of the experimental set-up for volatilisation measurement. The chromia pellet was put in a 4.6 cm diameter horizontal quartz tube. The exit end of the furnace tube was connected to a spiral condenser, which was further connected to a flask containing deionised water. The sample was exposed at the target temperatures of 873, 973 and 1073 K for 96 h. The average heating rate used in this work was 298.83 K min^−1^. The average cooling rate was 283 K min^−1^ from the target temperature to 773 K, and later the sample was naturally cooled down in the furnace to room temperature. For the gas mixture preparation, Ar mixed with 20%O_2_ or only O_2_ was purged into deionised water contained in a flask. The water was heated to 349 K to give the water vapour pressure of 0.4 bar calculated using standard thermodynamic data [41]. As a result, the exit gas from the flask was Ar–20%O_2_–40%H_2_O (by volume) or O_2_–40%H_2_O. The gas mixture flowed through the connecting tube covered by thermal insulator to avoid possible condensation. The gas flow rate was set at 1.1 dm^3^ min^−1^ at room temperature, corresponding to the linear gas velocity of 1.0 cm s^−1^ at that temperature. After the volatilisation test, the horizontal quartz tube and the spiral condenser were cleaned with 0.1 mol dm^−3^ HCl solution. The collected solution from the quartz tube and condenser cleaning was mixed with the condensate. This solution was further analysed by an inductively coupled plasma mass spectrometry analysis (Agilent 7900 ICP-MS) (Aligent Technologies, Santa Clara, CA, USA) to determine the amount of chromium volatilised from the chromia pellet.

Based on the experimental data from volatilisation measurement, we can further determine the standard enthalpy and entropy of Cr species volatilisation in humidified oxygen at 298 K. According to the chromium oxide volatilisation described in Reaction (1), Young and Pint [30] presented the equilibrium constant of that reaction as a function of gaseous partial pressures of oxygen, water vapour and CrO_2_(OH)_2_ volatile species. This expression can be re-arranged to be the following equation.

(15) $${\underline{p}}_{v}=\exp\left(-\frac{\Delta_{r}G_{\left(1\right)}^{\circ }}{RT}\right){\underline{p}}_{O_{2}}^{3/4}{\underline{p}}_{H_{2}O}$$

Here, ${\underline{p}}_{v}$ is the partial pressure of gas A divided by the pressure of gas A at standard state ($P_{A}^{\circ }$ which is set to be 1 bar). $\Delta_{r}G_{\left(1\right)}^{\circ }$ is the standard Gibbs free energy of Reaction (1). It is equal to the partial derivative of Gibbs free energy of the reacting system, i.e., the volatilisation reaction according to Reaction (1), with respect to the extent of reaction when all reacting species are in standard states at constant pressure and at a given temperature [42]. $\Delta_{r}G_{\left(1\right)}^{\circ }$ equals $\Delta_{r}H_{\left(1\right)}^{\circ }-T\Delta_{r}S_{\left(1\right)}^{\circ }$, where $\Delta_{r}H_{\left(1\right)}^{\circ }$ and $\Delta_{r}S_{\left(1\right)}^{\circ }$ are standard enthalpy and entropy of Reaction (1). They are defined in the similar manner as $\Delta_{r}G_{\left(1\right)}^{\circ }$, i.e., $\Delta_{r}H_{\left(1\right)}^{\circ }$ and $\Delta_{r}S_{\left(1\right)}^{\circ }$ are the partial derivatives of enthalpy and entropy of the reacting system, respectively, where Reaction (1) takes place with respect to the extent of reaction at constant pressure and temperature [42,43]. The standard state of solid species in Reaction (1), i.e., Cr_2_O_3_, is pure solid at standard pressure, i.e., 1 bar [42]. For each gas in Reaction (1), its standard state is the state that such gas is pure and exhibits the ideal gas behaviour at 1 bar [42].

${\underline{p}}_{v}$ can also be written as in Equation (16) to explicitly show the standard enthalpy of volatilisation according to Reaction (1) at 298 K ($\Delta_{r}H_{(1),298K}^{\circ }$) and the standard entropy of volatilisation by Reaction (1) at 298 K ($\Delta_{r}S_{(1),298K}^{\circ }$), with $\alpha_{T}$ described by Equations (17)–(19). The $c_{p}$ denotes the specific heat capacity at constant pressure of the interested species (J mol ^–1^ K^–1^).

(16) $${\underline{p}}_{v}=\alpha_{T}\exp\left(-\frac{\Delta_{r}H_{(1),298K}^{\circ }}{RT}\right)\exp\left(\frac{\Delta_{r}S_{(1),298K}^{\circ }}{R}\right){\underline{p}}_{O_{2}}^{3/4}{\underline{p}}_{H_{2}O}$$

(17) $$\alpha_{T}=\exp\left(-\frac{\Delta H_{T}}{RT}+\frac{\Delta S_{T}}{R}\right)$$

(18) $$\Delta H_{T}=\int_{298}^{T}(-0.5c_{{p,\mathrm{Cr}}_{2}O_{3}}-0.75c_{{p,O}_{2}}-c_{{p,H}_{2}O}+c_{{p,\mathrm{CrO}}_{2}{\left(\mathrm{OH}\right)}_{2}})dT$$

(19) $$\Delta S_{T}=\int_{298}^{T}(-0.5c_{{p,\mathrm{Cr}}_{2}O_{3}}-0.75c_{{p,O}_{2}}-c_{{p,H}_{2}O}+c_{{p,\mathrm{CrO}}_{2}{\left(\mathrm{OH}\right)}_{2}})T^{-1}dT$$

With the help of Equation (16), we proposed to express Equation (4) in the form shown in Equation (20). It can be seen from this equation that if $\ln(Tj_{\mathrm{Cr}}/k_{m}\alpha_{T})$ is plotted as a function of (1/R*T*), the negative slope of this plot is $\Delta_{r}H_{(1),298K}^{\circ }$, and the y-intercept (C_1_) includes $\Delta_{r}S_{(1),298K}^{\circ }$. Thus, by drawing this plot, we can obtain $\Delta_{r}H_{(1),298K}^{\circ }$ and $\Delta_{r}S_{(1),298K}^{\circ }$ from data of mass fluxes of Cr loss due to volatilisation that we measured from the experiment.

(20) $$\ln\left(\frac{T}{k_{m}\alpha_{T}}\cdot j_{\mathrm{Cr}}\right)=-\Delta_{r}H_{(1),298K}^{\circ }\cdot \frac{1}{RT}+C_{1}$$

(21) $$C_{1}=\frac{\Delta_{r}S_{(1),298K}^{\circ }}{R}+\ln\left(\frac{M_{\mathrm{Cr}}}{R}{\underline{p}}_{O_{2}}^{3/4}{\underline{p}}_{H_{2}O}p^{\circ }\right)$$

Surface of the pellet was observed by a scanning electron microscope (FEI QUANTA 450) (Thermo Fisher Scientific, Waltham, MA, USA). An X-ray diffractometer (Bruker AXS Model D8) (Bruker, Billerica, MA, USA) was used to identify the oxide phase using Cu Kα (α = 1.5406 Å). The obtained X-ray diffraction peaks were matched with standard patterns compiled by the International Centre for Diffraction Data (ICDD).

## 4. Results and Discussion

**Figure 3:**
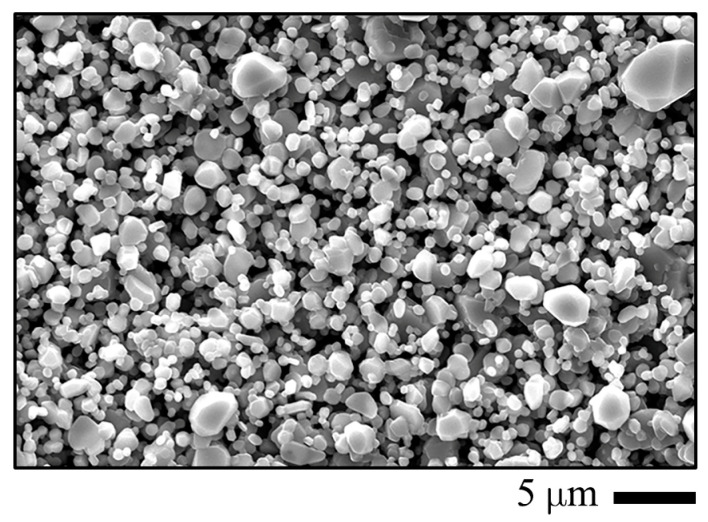
SEM image of the pellet surface.

**Figure 4:**
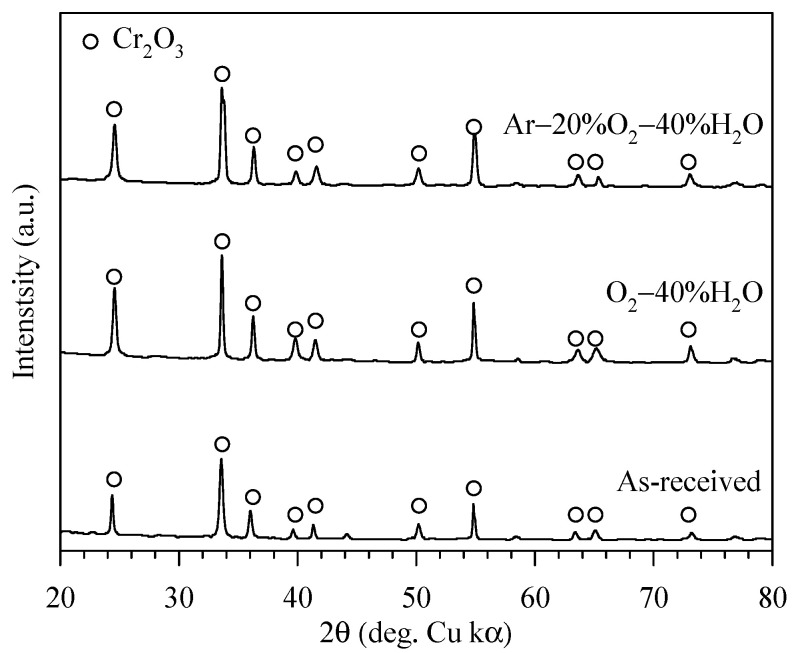
XRD results measured on the Cr_2_O_3_ pellet before and after exposure to Ar–20%O_2_–40%H_2_O and O_2_–40%H_2_O at 1073 K.

Figure 3 shows top-view images of the chromia pellet using the scanning electron microscopes. The images show smooth and crack-free surface and a dense sintered pellet. The XRD results in Figure 4 show that the detected peaks from the sintered pellet are only the peaks of Cr_2_O_3_ (ICDD 01-082-1484). After exposure in all studied conditions (Ar–20%O_2_–40%H_2_O and O_2_–40%H_2_O at 873, 973 and 1073 K), only Cr_2_O_3_ peaks were also detected as exemplified in Figure 4 for the samples exposed to both atmospheres at 1073 K.

Table 2 reports the measured mass fluxes of Cr loss due to volatilisation of Cr_2_O_3_ exposed to Ar–20%O_2_–40%H_2_O and O_2_–40%H_2_O at 873–1073 K. The experiment was repeated twice for each condition. The results show that the mass fluxes of Cr loss due to volatilisation are in the range of 1.0 × 10^−10^–1.3 × 10^−9^ g cm^−2^ s^−1^. In each atmosphere, the increased temperature increases the mass flux of Cr loss due to volatilisation. At each temperature, the mass flux of Cr loss due to volatilisation from the sample exposed to O_2_–40%H_2_O tends to be higher than the mass flux of Cr loss due to volatilisation from the sample exposed to Ar–20%O_2_–40%H_2_O.

### 4.1. Determination of Standard Enthalpy and Entropy of Cr Species Volatilisation in Humidified Oxygen at 298 K

**Figure 5:**
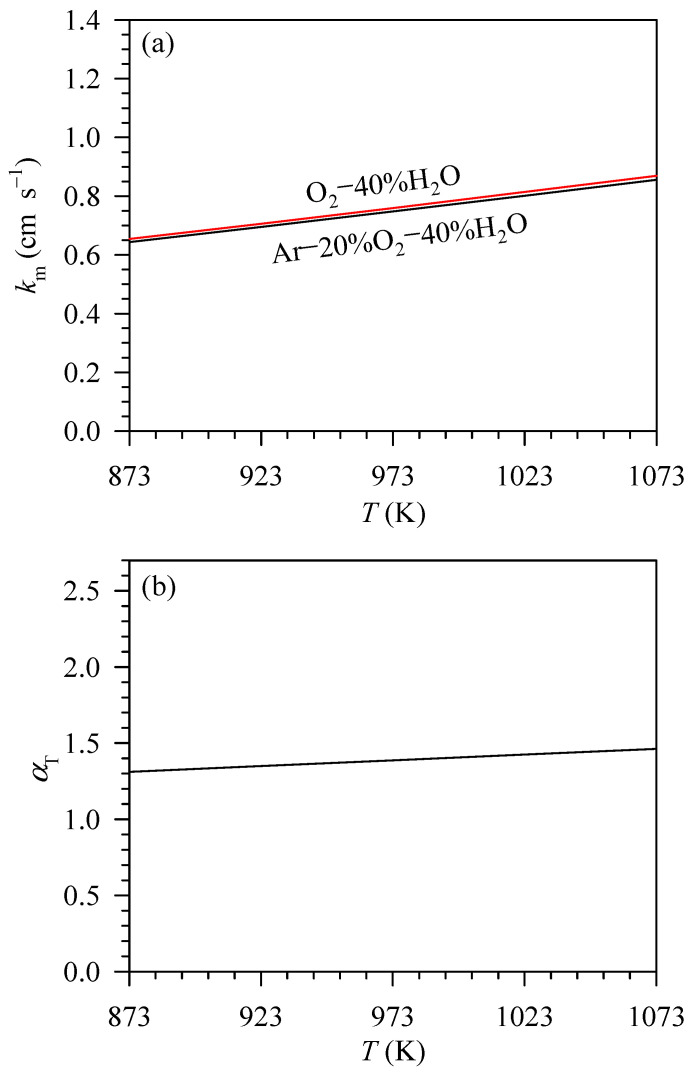
(**a**) Mass transfer coefficients of CrO_2_(OH)_2_ through Ar–20%O_2_–40%H_2_O and O_2_–40%H_2_O gas mixture and (**b**) the plot of $\alpha_{T}$ as a function of temperature at 873–1073 K.

To determine the standard enthalpy and entropy of Cr species volatilisation in humidified oxygen at 298 K according to Equations (20) and (21), the $j_{\mathrm{Cr}}$, $k_{m}$ and $\alpha_{T}$ values must be known. The $j_{\mathrm{Cr}}$ values were experimentally obtained as reported in Table 2. The $k_{m}$ values can be determined by Equations (5)–(14). It is noted that Equation (7) is used to calculate the binary gas diffusivity for low-density gases [36]. Bird et al. [36] applied Equation (7) to calculate the binary gas diffusivity of the CO–CO_2_ gas mixture at 296.1 K and 1 atm, implying that such gas mixture is considered as the low-density one. It can be calculated that the densities of CO-CO_2_ gas mixtures are about 10^−3^ g cm^−3^. In this study, at 1073 K and 1 atm, the densities of CrO_2_(OH)_2_–Ar, CrO_2_(OH)_2_–O_2_ and CrO_2_(OH)_2_–H_2_O are about 10^−4^ g cm^−3^, which are lower than densities of the CO–CO_2_ mixtures. Thus, the low-density behaviour of the CrO_2_(OH)_2_–Ar, CrO_2_(OH)_2_–O_2_ and CrO_2_(OH)_2_–H_2_O gas mixtures may be assumed, and, consequently, Equation (7) can be applied to calculate the diffusivities for those mixtures. The calculation shows that the $k_{m}$ values are in the range of 0.643–0.869 cm s^−1^ at 873–1073 K for the exposure to the studied atmospheres as illustrated in Figure 5a. It can be observed that, in each atmosphere, increasing temperature slightly changes the $k_{m}$ value. At each temperature, the $k_{m}$ value for the O_2_–40%H_2_O atmosphere is only slightly higher than the $k_{m}$ value for the Ar–20%O_2_–40%H_2_O atmosphere.

The $\alpha_{T}$ values were obtained by Equations (17)–(19) using the specific heat capacity of CrO_2_(OH)_2_ reported by Bauschlicher et al. [24] and specific heat capacities of other species (Cr_2_O_3_, O_2_ and H_2_O) reported by Kubaschewski et al. [41]. The $\alpha_{T}$ values are found to slightly increase from 1.311 to 1.462 when the temperature increases from 873 to 1073 K as shown in Figure 5b.

**Figure 6:**
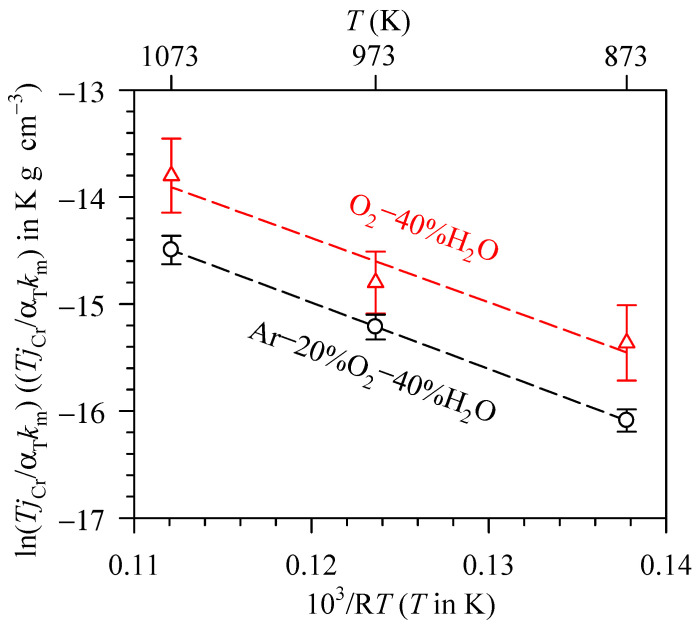
The plot to determine standard enthalpy and entropy of volatilisation at 298 K.

With the known $j_{\mathrm{Cr}}$, $k_{m}$ and $\alpha_{T}$ values, $\ln(Tj_{\mathrm{Cr}}/k_{m}\alpha_{T})$ can then be plotted as a function of (1/RT) according to Equation (20), giving rise to Figure 6. For Ar–20%O_2_–40%H_2_O, the $\ln(Tj_{\mathrm{Cr}}/k_{m}\alpha_{T})$ values are −15.4 ± 0.4, −14.8 ± 0.3 and −13.8 ± 0.4 K g cm^−3^ at 873, 973 and 1073 K, respectively. They are −16.1 ± 0.1, −15.2 ± 0.1 and −14.5 ± 0.1 K g cm^−3^ at 873, 973 and 1073 K for O_2_–40%H_2_O, respectively.

The experimental results presented in Figure 6 exhibit the linear relationship between $\ln(Tj_{\mathrm{Cr}}/k_{m}\alpha_{T})$ and (1/RT). These results are in agreement with the theoretical analysis in Equation (20) in the manner that when $\ln(Tj/\mathrm{Cr}k_{m}\alpha_{T})$ is plotted as a function of (1/RT), the linear relation between these two terms can be obtained. In addition, the slope of this plot is $-\Delta_{r}H_{(1),298K}^{\circ }$ and the y-intercept includes the terms $\Delta_{r}S_{(1),298K}^{\circ }$ as described by Equations (20) and (21). The $\Delta_{r}H_{(1),298K}^{\circ }$ values are found to be 62.1 and 60.1 kJ mol^−1^ for the measurement in Ar–20%O_2_–40%H_2_O and O_2_–40%H_2_O, giving the average value of 61.1 ± 1.0 kJ mol^−1^. When comparing with the literature, the standard enthalpy of Reaction (1) is calculated at 49.2 kJ mol^−1^ using the standard enthalpy of CrO_2_(OH)_2_ formation at 298 K ($\Delta_{f}H_{{\mathrm{CrO}}_{2}{\left(\mathrm{OH}\right)}_{2},298K}^{\circ }$) reported by Opila et al. [22] and other necessary data from the standard database [41]. By using the recent value of $\Delta_{f}H_{{\mathrm{CrO}}_{2}{\left(\mathrm{OH}\right)}_{2},298K}^{\circ }$ reported by Bauschlicher et al. [24] instead of the former one [22], the standard enthalpy of Reaction (1) is calculated at 58.1 kJ mol^−1^ and found to be closer to the one obtained from this work (61.1 kJ mol^−1^). For the $\Delta_{r}S_{(1),298K}^{\circ }$ determination, the values are found to be −41.1 and −44.9 J K^−1^ mol^−1^ for the measurement in Ar–20%O_2_–40%H_2_O and O_2_–40%H_2_O, respectively, giving the average value of −43.0 ± 1.9 J K^−1^ mol^−1^. This reaction entropy is calculated at −45.1 J K^−1^ mol^−1^ using the molar entropy of CrO_2_(OH)_2_ at 298 K (${\underline{S}}_{{\mathrm{CrO}}_{2}{\left(\mathrm{OH}\right)}_{2},298K}^{\circ }$) from Opila et al. [22] and other necessary data from Kubaschewski et al. [41]. When using ${\underline{S}}_{{\mathrm{CrO}}_{2}{\left(\mathrm{OH}\right)}_{2},298K}^{\circ }$ reported by Bauschlicher et al. [24] instead of the former one [22], the $\Delta_{r}S_{(1),298K}^{\circ }$ value is calculated at −43.6 J K^−1^ mol^−1^, which is very close to the one reported in the present work (−43.0 J K^−1^ mol^−1^).

To tabulate our measured data in the thermodynamic database, the standard enthalpy of CrO_2_(OH)_2_ formation at 298 K and 1 bar ($\Delta_{f}H_{{\mathrm{CrO}}_{2}{\left(\mathrm{OH}\right)}_{2},298K}^{\circ }$) and the molar entropy of CrO_2_(OH)_2_ at the standard state and at 298 K and 1 bar (${\underline{S}}_{{\mathrm{CrO}}_{2}{\left(\mathrm{OH}\right)}_{2},298K}^{\circ }$) are further calculated. As guided by Atkins and de Paula [44], $\Delta_{f}H_{{\mathrm{CrO}}_{2}{\left(\mathrm{OH}\right)}_{2},298K}^{\circ }$ is defined as the standard enthalpy of reaction to form one mole of CrO_2_(OH)_2_ from Cr_(s)_, O_2(g)_ and H_2(g)_ at 298 K and 1 bar. It is calculated using the $\Delta_{r}H_{(1),298K}^{\circ }$ value obtained from the present work and the other necessary data from Kubaschewski et al. [41], giving the value of −748.1 ± 1.0 J mol^−1^. This value differs from the one reported by Opila et al. (−760 ± 7 J mol^−1^) [22] by about 1.6%, and is very close to the one reported by Bauschlicher et al. (−751.10 J mol^−1^) [24], with a difference of about 0.4%. The ${\underline{S}}_{{\mathrm{CrO}}_{2}{\left(\mathrm{OH}\right)}_{2},298K}^{\circ }$ value is also calculated from the $\Delta_{r}S_{(1),298K}^{\circ }$ value obtained from the present work and the molar entropies of Cr_2_O_3_, O_2_ and H_2_O at 298 K from the standard database [41], giving the value of 340.1 ± 1.9 J K^−1^ mol^−1^. This value is very slightly different from the ${\underline{S}}_{{\mathrm{CrO}}_{2}{\left(\mathrm{OH}\right)}_{2},298K}^{\circ }$ value reported by Opila et al. (338 ± 16 J K^−1^ mol^−1^) [22] by about 0.6% and almost identical to the value recently reported by Bauschlicher et al. (339.5 J K^−1^mol^−1^) [22,24].

It can be seen in this section that the Cr species volatilisation measurement accompanied by the analysis using the transport theory of multicomponent gas mixture as proposed in this work can lead to the determination of the $\Delta_{f}H_{{\mathrm{CrO}}_{2}{\left(\mathrm{OH}\right)}_{2},298K}^{\circ }$ and ${\underline{S}}_{{\mathrm{CrO}}_{2}{\left(\mathrm{OH}\right)}_{2},298K}^{\circ }$ values similar to the ones obtained from the quantum calculations [24] and the measurement analysed using thermodynamic concepts [22]. Thus, the method proposed in this work should be considered as an alternative for determining thermodynamic quantities concerning the Cr species volatilisation, i.e., $\Delta_{r}H_{(1),298K}^{\circ }$, $\Delta_{r}S_{(1),298K}^{\circ }$, $\Delta_{f}H_{{\mathrm{CrO}}_{2}{\left(\mathrm{OH}\right)}_{2},298K}^{\circ }$ and ${\underline{S}}_{{\mathrm{CrO}}_{2}{\left(\mathrm{OH}\right)}_{2},298K}^{\circ }$.

### 4.2. Assessment of Thermodynamic Data Taken from Different Sources on the Calculated Mass Flux of Cr Loss Due to Volatilisation

**Figure 7:**
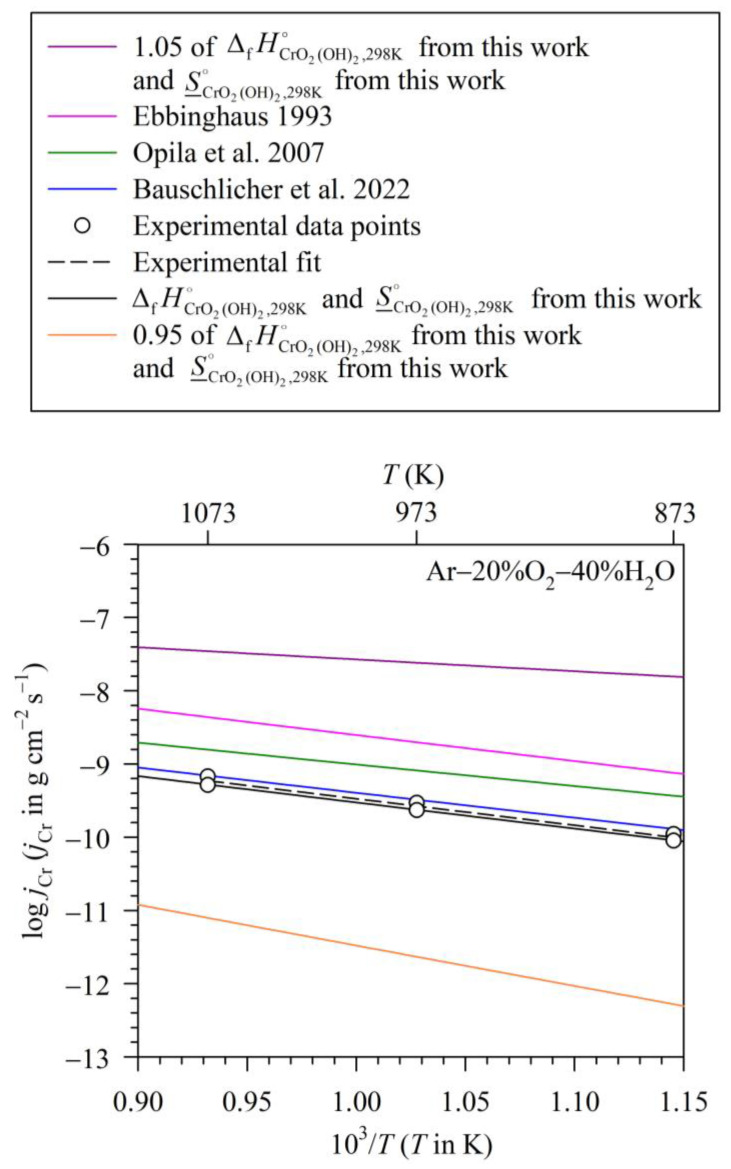
Measured and calculated mass fluxes of Cr loss due to volatilisation in logarithmic scale as a function of reciprocal temperature for Cr_2_O_3_ exposed to Ar–20%O_2_–40%H_2_O (For the calculated fluxes, the $\Delta_{f}H_{{\mathrm{CrO}}_{2}{\left(\mathrm{OH}\right)}_{2},298K}^{\circ }$ and ${\underline{S}}_{{\mathrm{CrO}}_{2}{\left(\mathrm{OH}\right)}_{2},298\;K}^{\circ }$ values are taken from various sources as cited in the box above the figure [22,24,33], while the $\Delta_{f}H_{298\;K}^{\circ }$, ${\underline{S}}_{298\;K}^{\circ }$ and $c_{p}$ values of Cr_2_O_3_, O_2_ and H_2_O are from Kubaschewski et al. [41] and the $c_{{p,\mathrm{CrO}}_{2}{\left(\mathrm{OH}\right)}_{2}}$ value is from Bauschlicher et al. [24]).

**Figure 8:**
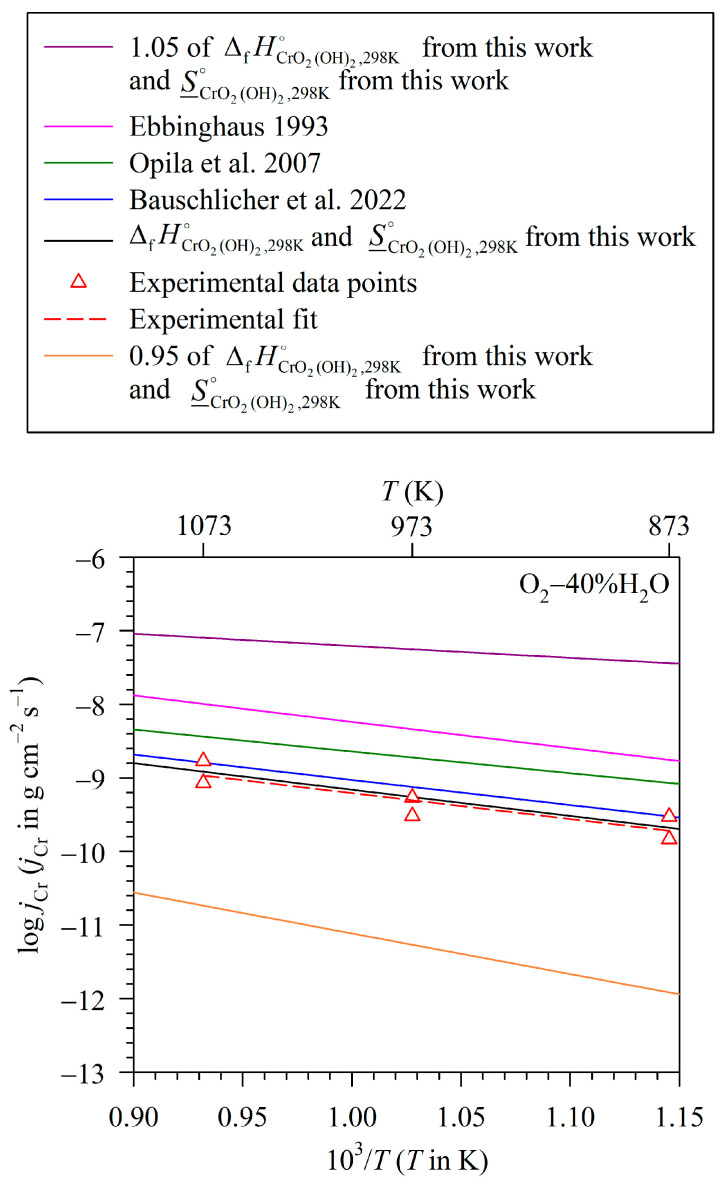
Measured and calculated mass fluxes of Cr loss due to volatilisation in logarithmic scale as a function of reciprocal temperature for Cr_2_O_3_ exposed to O_2_–40%H_2_O (For the calculated fluxes, the $\Delta_{f}H_{{\mathrm{CrO}}_{2}{\left(\mathrm{OH}\right)}_{2},298K}^{\circ }$ and ${\underline{S}}_{{\mathrm{CrO}}_{2}{\left(\mathrm{OH}\right)}_{2},298\;K}^{\circ }$ values are taken from various sources as cited in the box above the figure [22,24,33], while the $\Delta_{f}H_{298\;K}^{\circ }$, ${\underline{S}}_{298\;K}^{\circ }$ and $c_{p}$ values of Cr_2_O_3_, O_2_ and H_2_O are from Kubaschewski et al. [41] and the $c_{{p,\mathrm{CrO}}_{2}{\left(\mathrm{OH}\right)}_{2}}$ value is from Bauschlicher et al. [24]).

To assess the effect of thermodynamic data from various sources on the calculated mass flux of Cr loss due to volatilisation at various studied temperatures, the plot of mass flux of Cr loss due to volatilisation at different temperatures should be presented. It was observed as mentioned in the Introduction that the mass flux of Cr loss due to volatilisation in logarithmic scale tends to linearly relate with the absolute temperature in the cases of Fe-16Cr [34] and Fe-12Cr [31] exposed to humidified oxygen at high temperatures. Thus, Figure 7 and Figure 8 plot the mass flux of Cr loss due to volatilisation from the chromia pellet in a logarithmic scale as a function of the reciprocal temperature. The experimental data points are presented as circle symbols for the exposure to Ar–20%O_2_–40%H_2_O in Figure 7 and triangle symbols for the measurement in O_2_–40%H_2_O in Figure 8. The experimental fits between the mass fluxes of Cr loss due to volatilisation in logarithmic scale and inverse temperatures are also apparently found to be linear, drawn as dashed lines in these two figures. The other lines are constructed from the calculation using the data from the present work and different sources [22,24,33,41]. In constructing these lines, except for the $\Delta_{f}H_{{\mathrm{CrO}}_{2}{\left(\mathrm{OH}\right)}_{2},298K}^{\circ }$ and ${\underline{S}}_{{\mathrm{CrO}}_{2}{\left(\mathrm{OH}\right)}_{2},298K}^{\circ }$ values that are taken from various sources [22,24,33], data of standard enthalpies of Cr_2_O_3_, O_2_ and H_2_O formations at 298 K and the molar entropies of Cr_2_O_3_, O_2_ and H_2_O are from Kubaschewski et al. [41]. The specific heat capacities of Cr_2_O_3_, O_2_ and H_2_O are also from Kubaschewski et al. [41], while that of CrO_2_(OH)_2_ is from Bauschlicher et al. [24]. It is seen that the calculated lines in these two figures are also apparently linear.

**Figure 9:**
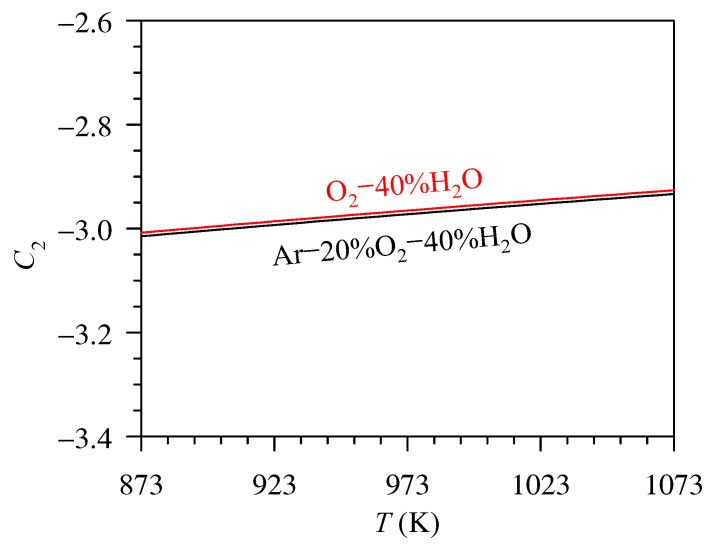
The plot of C_2_ as a function of temperature.

To explain the Arrhenius-like behaviour of each line in Figure 7 and Figure 8, we proposed to re-arrange Equation (20) to the form of Equation (22), where β consists of the temperature-independent term (i.e., C_1_/ln10) and the temperature-dependent term (C_2_). Figure 9 presents the C_2_ value as a function of temperature. It can be seen that the C_2_ value increases only about 2.7% when the temperature rises from 873 to 1073 K, i.e., 2.69 and 2.71% for the measurement in Ar–20%O_2_–40%H_2_O and O_2_–40%H_2_O, respectively. With a slight variation in the C_2_ value with temperature, the *β* value according to Equation (23) can then be estimated to be constant, and, consequently, Equation (22) can be approximated to follow the Arrhenius relation. However, it should be noted that the mass flux of Cr loss due to volatilisation and absolute temperature is likely to follow the Arrhenius-like form because of the mathematical reason, not because of the physico-chemical reason concerning the activation energy concept [31,45]. This is to say that if C_2_ is assumed to be unchanged with temperature, the slopes of the plots in Figure 7 and Figure 8 can lead to the determination of the standard enthalpy of volatilisation at 298 K, not the activation energy as classically obtained from the Arrhenius relation between the absolute temperature and the reaction rate constant [31,45].

(22) $$l\mathrm{og}\;j_{\mathrm{Cr}}=-\frac{\Delta_{r}H_{(1),298K}^{\circ }}{(\ln10)R}\cdot \frac{1}{T}+\beta$$

(23) $$\beta =\frac{C_{1}}{\ln10}+C_{2}$$

(24) $$C_{2}=\log\left(\frac{k_{m}}{T}\right)-\frac{\Delta H_{T}}{(\ln10)RT}+\frac{\Delta S_{T}}{(\ln10)R}$$

After understanding the reason for the approximately linear characteristics of the plots in Figure 7 and Figure 8, the assessment of thermodynamic data from various sources on the calculated mass flux of Cr loss due to volatilisation is further considered. The first line to be discussed is the one constructed using data reported in the present work, i.e., $\Delta_{f}H_{{\mathrm{CrO}}_{2}{\left(\mathrm{OH}\right)}_{2},298K}^{\circ }$ = −748.1 ± 1.0 kJ mol^−1^ and ${\underline{S}}_{{\mathrm{CrO}}_{2}{\left(\mathrm{OH}\right)}_{2},298K}^{\circ }$ = 340.1 ± 1.9 J K^−1^ mol^−1^. It can be seen in Figure 7 and Figure 8 that the calculated lines almost coincide with the ones obtained from the experimental fit in both studied atmospheres. When comparing with the data reported by Bauschlicher et al. [24], as discussed in the former section, the $\Delta_{f}H_{{\mathrm{CrO}}_{2}{\left(\mathrm{OH}\right)}_{2},298K}^{\circ }$ value reported by them [24] is close to the one reported in this work, with a difference only of about 0.4%, while their ${\underline{S}}_{{\mathrm{CrO}}_{2}{\left(\mathrm{OH}\right)}_{2},298K}^{\circ }$ value (339.5 J K^–1^ mol^–1^) [24] is almost identical to the one reported here (340.1 ± 1.9 J K^−1^ mol^−1^). As a consequence, the calculated line of mass flux of Cr loss due to volatilisation using $\Delta_{f}H_{{\mathrm{CrO}}_{2}{\left(\mathrm{OH}\right)}_{2},298K}^{\circ }$ and ${\underline{S}}_{{\mathrm{CrO}}_{2}{\left(\mathrm{OH}\right)}_{2},298K}^{\circ }$ data from Bauschlicher et al. [24] is found to lie close to the line calculated from the data obtained from this work. In fact, it is seen in Figure 7 and Figure 8 that these two calculated lines lie within the experimental error of the volatilisation measurement.

On the contrary, it was observed that even though the ${\underline{S}}_{{\mathrm{CrO}}_{2}{\left(\mathrm{OH}\right)}_{2},298K}^{\circ }$ value is changed by about 5%, this difference can have a considerable effect on the calculated mass flux of Cr loss due to volatilisation. For example, though the $\Delta_{f}H_{{\mathrm{CrO}}_{2}{\left(\mathrm{OH}\right)}_{2},298K}^{\circ }$ reported by Ebbinghaus (−748.3 ± 4.3 J mol^−1^) [33] is almost identical to the one reported in the present work (−748.1 ± 1.9 J mol^−1^), their ${\underline{S}}_{{\mathrm{CrO}}_{2}{\left(\mathrm{OH}\right)}_{2},298K}^{\circ }$ value (357.4 ± 4 J K^−1^ mol^−1^) [33] is about 5% higher than the value reported in this work. Consequently, it is found that the mass fluxes of Cr loss due to volatilisation calculated using the ${\underline{S}}_{{\mathrm{CrO}}_{2}{\left(\mathrm{OH}\right)}_{2},298K}^{\circ }$ and $\Delta_{f}H_{{\mathrm{CrO}}_{2}{\left(\mathrm{OH}\right)}_{2},298K}^{\circ }$ data reported by Ebbinghaus [33] are in the range of 7.3 × 10^−10^ to 4.2 × 10^−9^ g cm^−2^ s^−1^ for the exposure to Ar–20%O_2_–40%H_2_O at 873–1073 K, while the mass fluxes of Cr loss due to volatilisation calculated using ${\underline{S}}_{{\mathrm{CrO}}_{2}{\left(\mathrm{OH}\right)}_{2},298K}^{\circ }$ and $\Delta_{f}H_{{\mathrm{CrO}}_{2}{\left(\mathrm{OH}\right)}_{2},298K}^{\circ }$ data from this work are in the range of 8.8 × 10^−11^ to 5.1 × 10^−10^ g cm^−2^ s^−1^. The former mass fluxes of Cr loss due to volatilisation are therefore larger than the latter ones by the factor of about 10, i.e., by the factors of 8.2–8.3 for the tests at 873–1073 K. The comparative values between these two fluxes are also similar for the results in O_2_–40%H_2_O. To explain these results graphically, it is seen that the increased ${\underline{S}}_{{\mathrm{CrO}}_{2}{\left(\mathrm{OH}\right)}_{2},298K}^{\circ }$ value increases $\Delta_{r}S_{(1),298\;K}^{\circ }$. The increased $\Delta_{r}S_{(1),298\;K}^{\circ }$ value increases the C_1_ value according to Equation (21), and therefore increases the β value according to Equation (23). This finally results in the increased mass flux of Cr loss due to volatilisation according to Equation (22), thus shifting the line of mass flux of Cr loss due to volatilisation to a more positive direction as observed in Figure 7 and Figure 8.

In another case, when ${\underline{S}}_{{\mathrm{CrO}}_{2}{\left(\mathrm{OH}\right)}_{2},298K}^{\circ }$ is unchanged or slightly different, the increase in the $\Delta_{f}H_{{\mathrm{CrO}}_{2}{\left(\mathrm{OH}\right)}_{2},298K}^{\circ }$ value can also have an effect on the calculated mass flux of Cr loss due to volatilisation. For example, the ${\underline{S}}_{{\mathrm{CrO}}_{2}{\left(\mathrm{OH}\right)}_{2},298K}^{\circ }$ value reported by Opila et al. [22] is only slightly different from the one reported here by about 0.6%; however, the $\Delta_{f}H_{{\mathrm{CrO}}_{2}{\left(\mathrm{OH}\right)}_{2},298K}^{\circ }$ value reported by the same authors [22] is less than the one reported in this work by about 1.6%. Consequently, the mass fluxes of Cr loss due to volatilisation calculated using $\Delta_{f}H_{{\mathrm{CrO}}_{2}{\left(\mathrm{OH}\right)}_{2},298K}^{\circ }$ and ${\underline{S}}_{{\mathrm{CrO}}_{2}{\left(\mathrm{OH}\right)}_{2},298K}^{\circ }$ data from Opila et al. [22] are in the range of 3.5 × 10^–10^ to 1.5 × 10^−9^ g cm^−2^ s^−1^ for the exposure to Ar–20%O_2_–40%H_2_O at 873–1073 K. These values are larger than the ones calculated using $\Delta_{f}H_{{\mathrm{CrO}}_{2}{\left(\mathrm{OH}\right)}_{2},298K}^{\circ }$ and ${\underline{S}}_{{\mathrm{CrO}}_{2}{\left(\mathrm{OH}\right)}_{2},298K}^{\circ }$ data from the present work by the factors of about 3 to 4. A similar effect is also observed for the results in O_2_–40%H_2_O.

To assess the effect of a larger difference in $\Delta_{f}H_{{\mathrm{CrO}}_{2}{\left(\mathrm{OH}\right)}_{2},298K}^{\circ }$ on the calculated mass flux of Cr loss due to volatilisation, we simulated the situation in which there is no change in ${\underline{S}}_{{\mathrm{CrO}}_{2}{\left(\mathrm{OH}\right)}_{2},298K}^{\circ }$. However, it is considered in such a situation that the value of the standard enthalpy of CrO_2_(OH)_2_ formation at 298 K is lower than the one reported in the present work by 5%, meaning that such value is $1.05\times \Delta_{f}H_{{\mathrm{CrO}}_{2}{\left(\mathrm{OH}\right)}_{2},298\;K}^{\circ }$, equalling 1.05 × (−748.1) or −785.5 J mol^−1^. It can be calculated that the mass fluxes of Cr loss due to volatilisation in this case are in the range of 1.6 × 10^−8^ to 3.5 × 10^−8^ g cm^−2^ s^−1^ for the exposure to Ar–20%O_2_–40%H_2_O at 873–1073 K. These values are larger than the mass fluxes of Cr loss due to volatilisation calculated using the $\Delta_{f}H_{{\mathrm{CrO}}_{2}{\left(\mathrm{OH}\right)}_{2},298K}^{\circ }$ and ${\underline{S}}_{{\mathrm{CrO}}_{2}{\left(\mathrm{OH}\right)}_{2},298K}^{\circ }$ data reported in the present work by the factors of 66–173 for the tests at 873–1073 K. A similar effect is also observed for the tests in O_2_–40%H_2_O. In the graphical aspect, the decrease in the $\Delta_{f}H_{{\mathrm{CrO}}_{2}{\left(\mathrm{OH}\right)}_{2},298K}^{\circ }$ value decreases the $\Delta_{r}H_{(1),298K}^{\circ }$ value or increases the $-\Delta_{r}H_{(1),298\;K}^{\circ }$ value, which is proportional to the slope of the plot in Figure 7 and Figure 8. With unchanged $\Delta_{f}S_{{\mathrm{CrO}}_{2}{\left(\mathrm{OH}\right)}_{2},298\;K}^{\circ }$, which means that the y-intercept of the plot in Figure 7 and Figure 8 is fixed, increasing the slope thus helps increase the mass flux of Cr loss due to volatilisation at a given temperature as observed in Figure 7 and Figure 8. In the opposite way, if the $\Delta_{f}H_{{\mathrm{CrO}}_{2}{\left(\mathrm{OH}\right)}_{2},298K}^{\circ }$ value is higher than the one reported in this work by 5% (i.e., its value is $0.95\times \Delta_{f}H_{{\mathrm{CrO}}_{2}{\left(\mathrm{OH}\right)}_{2},298\;K}^{\circ }$, equalling −710.7 J mol^−1^) and the ${\underline{S}}_{{\mathrm{CrO}}_{2}{\left(\mathrm{OH}\right)}_{2},298K}^{\circ }$ value is unchanged, the calculated mass fluxes of Cr loss due to volatilisation are in the range of 1.2 × 10^−12^ to 1.8 × 10^−11^ g cm^−2^ s^−1^ for the exposure to Ar–20%O_2_–40%H_2_O at 873–1073 K. The mass fluxes of Cr loss due to volatilisation calculated using the $\Delta_{f}H_{{\mathrm{CrO}}_{2}{\left(\mathrm{OH}\right)}_{2},298K}^{\circ }$ and ${\underline{S}}_{{\mathrm{CrO}}_{2}{\left(\mathrm{OH}\right)}_{2},298K}^{\circ }$ values reported in this work are larger than those values by the factors of 66–173 for the tests at 873–1073 K. A similar effect is also observed for the results in O_2_–40%H_2_O. These analyses mark the importance of the reliability of thermodynamic data reported in different sources in giving reliable values of the mass flux of Cr loss due to the volatilisation calculated from them.

## 5. Conclusions

A method to determine the standard volatilisation enthalpy and entropy of chromia exposed to humidified oxygen at a temperature of 298 K was proposed. The expression used for this determination was derived based on the transport theory of multicomponent gas mixtures and took into consideration the changes in the enthalpy and entropy of the volatilisation reaction with temperature. To determine these thermodynamic quantities, the Cr species volatilisation from chromia exposed to Ar–20%O_2_–40%H_2_O and O_2_–40%H_2_O atmospheres at 873–1073 K were primarily measured, giving values in the range of 1.0 × 10^−10^–1.3 × 10^−9^ g cm^−2^ s^−1^. By using the proposed expression, the standard enthalpy and entropy of volatilisation at 298 K were determined, giving the values of 61.1 ± 1.0 kJ mol^−1^ and −43.0 ± 1.9 J K^−1^ mol^−1^, respectively. The standard enthalpy of CrO_2_(OH)_2_ formation and the molar entropy of CrO_2_(OH)_2_ at 298 K were also derived, obtaining the values of −748.1 ± 1.0 kJ mol^−1^ and 340.1 ± 1.9 J K^−1^ mol^−1^, respectively.

In addition, the relation between the mass flux of Cr loss due to volatilisation in the logarithmic scale and the reciprocal temperature tended to be linear. Variations in the thermodynamic data used can significantly affect the calculated mass flux of Cr loss due to volatilisation or a graphical shift in the calculated line in the Arrhenius-like diagram. This diagram was then used to help evaluate the reliability of the standard enthalpy of CrO_2_(OH)_2_ formation and the molar entropy of CrO_2_(OH)_2_ at 298 K. The analysis showed that if the reported molar entropy of CrO_2_(OH)_2_ at 298 K or the standard enthalpy of CrO_2_(OH)_2_ formation at 298 K is varied by about 5%, the calculated mass flux of Cr loss due to volatilisation can be varied up to about 10 times or 170 times, respectively. Thus, the use of reliable thermodynamic data is important for the correct prediction of the mass flux of Cr loss due to volatilisation.

## Figures and Tables

**Table 1:** Molar masses (M), collision diameters (σ) and characteristic energies divided by the Boltzmann constant ($\varepsilon /k_{B}$) of gaseous species involving in the Cr species volatilisation in Ar-O_2_-H_2_O gas mixture [30,37,39].

Gaseous Species	$M_{A}$(g mol^–1^)		$\sigma_{A}$(Å)		$\varepsilon_{A}/k_{B}$(K)	
	Value	Ref.	Value	Ref.	Value	Ref.
CrO_2_(OH)_2_	118.01	[39]	4.5	[30]	340	[30]
Ar	39.9480	[39]	3.542	[37]	93.3	[37]
O2	31.9988	[39]	3.467	[37]	106.7	[37]
H2O	18.0152	[39]	2.641	[37]	809.1	[37]

**Table 2:** Mass fluxes of Cr loss due to volatilisation of Cr_2_O_3_ exposed to Ar–20%O_2_–40%H_2_O and O_2_–40%H_2_O.

Atmosphere	Temperature (K)	Mass Flux of Cr loss Due to Volatilisation (×10^−10^ g cm^−2^ s^−1^)	
		Average	Error
Ar–20%O_2_–40%H_2_O	873	1.0	0.1
	973	2.7	0.3
	1073	6.0	0.8
O_2_–40%H_2_O	873	2.2	0.8
	973	4.2	1.2
	1073	13	4.2

## Data Availability

The original contributions presented in the study are included in the article; further inquiries can be directed to the corresponding author.
